# In Vitro Phytotherapeutic Properties of Aqueous Extracted *Adenia viridiflora* Craib. towards Civilization Diseases

**DOI:** 10.3390/molecules26041082

**Published:** 2021-02-18

**Authors:** Werawat Wannasaksri, Nattira On-Nom, Chaowanee Chupeerach, Piya Temviriyanukul, Somsri Charoenkiatkul, Uthaiwan Suttisansanee

**Affiliations:** 1 Institute of Nutrition, Mahidol University, Salaya, Phuttamonthon, Nakhon Pathom 73170, Thailand; nit.frank@gmail.com (W.W.); nattira.onn@mahidol.ac.th (N.O.-N.); chaowanee.chu@mahidol.ac.th (C.C.); piya.tem@mahidol.ac.th (P.T.); somsri.chr@mahidol.ac.th (S.C.); 2 Food and Nutrition Academic and Research Cluster, Institute of Nutrition, Mahidol University, Salaya, Phuttamonthon, Nakhon Pathom 73170, Thailand

**Keywords:** *Adenia viridiflora* Craib, old leaves, young shoots, phenolic compositions, antioxidant activities, enzyme inhibitory activities

## Abstract

*Adenia viridiflora* Craib. is an indigenous edible plant that became an endangered species due to limited consumption of the local population with unknown reproduction and growth conditions. The plant is used as a traditional herb; however, its health applications lack scientific-based evidence. *A. viridiflora* Craib. plant parts (old leaves and young shoots) from four areas as Kamphaeng Phet (KP), Muang Nakhon Ratchasima (MN), Pakchong Nakhon Ratchasima (PN), and Uthai Thani (UT) origins were investigated for phenolic compositions and in vitro health properties through the inhibition of key enzymes relevant to obesity (lipase), diabetes (α-glucosidase and dipeptidyl peptidase-IV), Alzheimer’s disease (cholinesterases and β-secretase), and hypertension (angiotensin-converting enzyme). Phenolics including *p*-coumaric acid, sinapic acid, naringenin, and apigenin were detected in old leaves and young shoots in all plant origins. Old leaves exhibited higher total phenolic contents (TPCs) and total flavonoid contents (TFCs), leading to higher enzyme inhibitory activities than young shoots. Besides, PN and MN with higher TPCs and TFCs tended to exhibit greater enzyme inhibitory activities than others. These results will be useful to promote this plant as a healthy food with valuable medicinal capacities to support its consumption and agricultural stimulation, leading to sustainable conservation of this endangered species.

## 1. Introduction

*Adenia viridiflora* Craib. (or Pak E-noon in Thai) in the family Passifloraceae is a wild indigenous climbing plant that grows in mixed deciduous and deciduous dipterocarp forest areas in Northeastern Thailand. The smooth dark green young round vine turns rough and light brown when getting older, while dark green to violet-red shoots put out tendrils to climb on other trees. *A. viridiflora* Craib. is a monocot plant with heart-shaped leaves, which can extend to 5–8 cm in width and 8–15 cm in length. The leaves have a circular shaped organ at the base that is specific to this plant. The edible parts of the plant are the shoot, leaf, flower, and fruit, with a harvesting time between March and August. At other times of the year, the plant assumes a resting state due to unsuitable growing conditions.

*A. viridiflora* Craib. can be consumed fresh or as a fermented vegetable. The plant is also used as a traditional herb to treat urinary tract infections, fever, giddiness, fainting, and diarrhea [1]. However, consumption and health applications of *A. viridiflora* Craib. are limited to the local population with no scientific-based evidence. Several traditional applications as health benefits have been reported in other *Adenia* spp. with suggestions that asthma and fatigue symptoms could be reduced by *A. fruticosa* and *A. spinosa* [2], while a reduction in abdomen pain was promoted by *A. cardifolia* [3] and traditional application in infectious diseases, especially sexually transmitted diseases, was suggested in *A. lobata* [4]. Traditional use in malaria prevention, stomach cramps, and abortion as well as other applications with scientific proof including in vitro anticoagulant effect, antidepressant and anxiolytic activities in mice, in vitro anti-plasmodial activity, antimicrobial activity (*Bacillus polymyxa* and *Escherichia coli*), and anti-trypanocidal activity (*Trypanosoma congolesene*, *T. brucei*, and *T. congolense*) were reported in *A. cissampeloides* [5,6,7,8]. Scientific evidence for the medicinal applications was reported in *A. lobata*, including anti-hemorrhoidal activity, antimicrobial activities (Gram-negative bacilli), β-lactamases inhibition (degradation of antibiotics, β-lactams), and anti-parasitic activities (*Plasmodium falciparum*, *T. brucei*, *Leishmania donovani*, Nematoda, and Mites) [9,10,11,12,13]. Anti-plasmodial activity on *Plasmodium falciparum* was also reported in *A. gummifera* [14]. This information suggested that *A. viridiflora* Craib. might also possess medicinal applications. No information on bioactive compounds of *A. viridiflora* Craib. have previously been reported. Its reproduction and growth condition are also unknown, causing the plant to become an endangered species. *A. viridiflora* Craib. was, thus, registered as an endangered species in the Plant Genetic Conservation Project under the royal initiation of Her Royal Highness Princess Maha Chakri Sirindhorn (RSPG) in 2010.

Due to the lack of information on cultivation and health-promoting bioactivities, this research investigated the effects of edible plant parts (old leaves and young shoots) on phenolic compositions, antioxidant activities, and in vitro health properties of four originated *A. viridiflora* Craib. plants. Many parts of *A. viridiflora* Craib. can be consumed; however, old leaves (30–50 cm from the top) and young shoots (0–30 cm from the top) were selected due to their consumption popularity and availability. To investigate the effect of growth location on genetic variation for future agricultural purposes, four places of origin were selected as Kamphaeng Phet (KP), Muang Nakhon Ratchasima (MN), Pakchong Nakhon Ratchasima (PN), and Uthai Thani (UT) due to high populations of *A. viridiflora* Craib. growing naturally in these areas. The plants were collected and cultivated at the conservative plant area for experimentally botanical purposes at Khlong Phai, Sikhio district, Nakhon Ratchasima, Thailand. Due to the limited scientific-based evidence on health properties of *A. viridiflora* Craib., phenolic compositions, antioxidant activities, and inhibition of the key enzymes relevant to the occurrence of obesity (lipase), diabetes (α-glucosidase and dipeptidyl peptidase-IV (DPP-IV)), hypertension (angiotensin-converting enzyme (ACE)), and Alzheimer’s disease (acetylcholinesterase (AChE), butyrylcholinesterase (BChE), and β-secretase (BACE-1)) were also investigated. Knowledge gained from this research can be used to promote *A. viridiflora* Craib. as a healthy vegetable with valuable medicinal properties to both locals and outsiders to support its consumption and agricultural stimulation. Functional food development is also an interesting topic for future application of *A. viridiflora* Craib. Information from this research can lead to sustainable conservation and utilization of *A. viridiflora* Craib. to circumvent its extinction.

## 2. Results

### 2.1. Phenolic Profiles

Results from high performance liquid chromatography (HPLC) suggested that *A. viridiflora* Craib. plants from the four growing areas Kamphaeng Phet (KP), Muang Nakhon Ratchasima (MN), Pakchong Nakhon Ratchasima (PN), and Uthai Thani (UT) contained phenolic acids including *p*-coumaric acid and sinapic acid, and flavonoids including naringenin and apigenin (Table 1). The most abundant phenolic acid, *p*-coumaric acid, was found in the range of 22.60–97.09 mg/100 g dry weight (DW), with significantly higher contents detected in old leaves (55.86–97.09 mg/100 g DW) than in young shoots (22.60–38.98 mg/100 g DW). Both old leaves and young shoots from MN contained the highest *p*-coumaric acid content, while PN gave the lowest. Sinapic acid was found in the range of 4.49–14.00 mg/100 g DW, with significantly higher contents detected in old leaves (8.60–14.00 mg/100 g DW) than young shoots (4.49–7.19 mg/100 g DW). Old leaves and young shoots of PN and MN contained higher contents of sinapic acid than in the other areas. Interestingly, caffeic acid was also detected in *A. viridiflora* Craib. in a range 2.21–11.79 mg/100 g DW. However, this phenolic acid only presented in old leaves of KP and UT and young shoots of UT, with significantly higher contents detected in old leaves than in young shoots of UT. The most abundant flavonoid, naringenin, were in the range 1957.50–2164.95 mg/100 g DW, with insignificantly different contents of naringenin observed between old leaves and young shoots in all plant samples. Apigenin was found in the range 1.22–10.60 mg/100 g DW. Old leaves of MN and PN contained higher apigenin contents than their young shoots. However, opposite results were observed in KP and UT, where young shoots contained higher apigenin contents than old leaves. Among all plant sources with the same plant parts, MN contained the highest apigenin contents in old leaves, while KP contained the highest contents in young shoots.

Total phenolic contents (TPCs) were in the range 20.38–28.70 mg gallic acid equivalent (GAE)/g DW, suggesting that TPCs in old leaves of PN and KP were higher than the others, while MN exhibited the highest TPCs in young shoots (Table 1). Old leaves contained significantly higher TPCs than young shoots, with the exception of MN, where insignificant differences in TPCs were observed between old leaves and young shoots. Interestingly, both old leaves and young shoots of PN contained higher total flavonoid contents (TFCs) than the other plant samples. Old leaves of all plants contained significantly higher TFCs than young shoots, with the exception of KP, where opposite results were observed. Old leaves of KP contained significantly lower TFCs than young shoots.

### 2.2. Antioxidant Activities

Antioxidant activities of *A. viridiflora* Craib. were investigated using 2,2-diphenyl-1-picrylhydrazyl (DPPH) radical scavenging, ferric reducing antioxidant power (FRAP), and oxygen radical antioxidant capacity (ORAC) assays (Table 2). DPPH radical scavenging activities were in the range 0.96–1.44 µmol Trolox equivalent (TE)/100 g DW. Old leaves of all plants exhibited significantly lower free radical scavenging activities than young shoots, with the exception of PN, where old leaves exhibited significantly higher scavenging activities than young shoots. For the same plant parts of all origins, old leaves of PN and young shoots of MN significantly exhibited the highest scavenging activities. Antioxidant activities analyzed by FRAP assay were in the range 14.36–37.00 µmol TE/g DW. Old leaves of all plants exhibited higher reducing activities than young shoots. Old leaves of all plants exhibited similar reducing activities while reducing activities in young shoots of MN and PN were higher than in the other areas of origin. For ORAC assay, antioxidant activities were in the range 753.77–1438.85 µmol TE/g DW. Antioxidant activities in old leaves of MN were higher than in young shoots, while opposite results were observed in KP, where young shoots exhibited higher ORAC activities than old leaves. ORAC activities in old leaves and young shoots of PN and UT were insignificantly different. Interestingly, both old leaves and young shoots of PN exhibited higher ORAC activities than the other plant samples.

### 2.3. Enzyme Inhibitory Activities

Enzyme inhibitory activities were analyzed using extracts of both old leaves, and young shoots of the four originated *A. viridiflora* Craib. plants to investigate the possibility that these extracts could control the occurrence of obesity (lipase), diabetes (α-glucosidase and dipeptidyl peptidase-IV), Alzheimer’s disease (cholinesterases and β-secretase), and hypertension (angiotensin-converting enzyme) through inhibition of the key enzymes. Results suggested that the extracts could inhibit the enzyme that controls lipid degradation, lipase, in the range 8.41–23.54% inhibition using an extract concentration of 10 mg/mL (Table 3). Old leaves exhibited significantly higher lipase inhibitory activities than young shoots in all plants. For the same plant part, old leaves and young shoots of MN and PN exhibited higher lipase inhibitory activities than the other origins.

To investigate the potential of *A. viridiflora* Craib. extracts to control diabetes, the inhibitory activities of α-glucosidase, the carbohydrate degrading enzyme, and dipeptidyl peptidase-IV (DPP-IV), the enzyme that controls the release of insulin, were examined. Inhibitions of α-glucosidase ranged 58.08%–82.82% using the extract concentration of 12.5 mg/mL (Table 3). Comparing between plant parts, young shoots of all plants exhibited higher α-glucosidase inhibitory activities than their corresponding old leaves, with the exception of MN, which showed insignificant differences in inhibitory activities between old leaves and young shoots. Old leaves of PN and young shoots of PN and KP exhibited higher α-glucosidase inhibitory activities than the other origins with the same plant part. Inhibition against DPP-IV ranged 30.19–74.14% using the extract concentration of 1.25 mg/mL (Table 3). Old leaves of all origins exhibited significantly higher DPP-IV inhibitory activities than their corresponding young shoots, with the exception of MN, where young shoots exhibited significantly higher inhibitory activities than old leaves. Old leaves of PN and young shoots of MN exhibited higher inhibitory activities than plants from the other three areas with the same plant part.

Degradation of neurotransmitters by cholinesterases (ChEs), including acetylcholinesterase (AChE) and butyrylcholinesterase (BChE), have been hypothesized as a cause of Alzheimer’s disease (AD) occurrence. Inhibitory activities against AChE of *A. viridiflora* Craib. extracts ranged 16.29–28.68% using the extract concentration of 10 mg/mL (Table 3). Insignificant differences in AChE inhibitory activities between old leaves and young shoots were observed in all plants, with the exception of PN, where old leaves exhibited significantly higher inhibitory activities than young shoots. Old leaves and young shoots of KP exhibited significantly higher AChE inhibitory activities than plants from the other areas with the same plant part. Lower inhibitory activities against BChE (5.18–18.70%) than AChE were observed using the same extract concentration (Table 3). Higher BChE inhibitory activities were detected in old leaves than young shoots, while old leaves and young shoots of MN exhibited significantly higher inhibitory activities than plants from the other areas with the same plant parts. Another hypothesis for AD occurrence is the formation of amyloid plaque by β-secretase (BACE-1). Inhibitory activities against BACE-1 ranged 70.49–90.63% using the extract concentration of 1.5 mg/mL (Table 3). Insignificant differences in BACE-1 inhibitory activities between old leaves and young shoots of plants from all areas were observed, with the exception of PN, where old leaves exhibited significantly higher inhibitory activities than young shoots.

Hypertension can be controlled via inhibition of angiotensin-converting enzyme (ACE), the key enzyme in renin-angiotensin-aldosterone (RAAS) hormonal cascade. Results suggested that *A. viridiflora* Craib. extracts exhibited ACE inhibitory activities in the range 47.84%–65.53% using the extract concentration of 0.08 mg/mL (Table 3). Insignificant differences in ACE inhibitory activities between old leaves and young shoots of all plants were observed, with the exception of MN, where young shoots exhibited significantly higher inhibitory activities than old leaves. Old leaves of MN and PM and young shoots of MN exhibited significantly higher ACE inhibitory activities than plants from the other areas.

## 3. Discussion

*Adenia viridiflora* Craib. is a wild indigenous climbing plant found in Northeastern Thailand that is consumed by some locals without any scientific-based health evidence. The plant was registered as an endangered species in the Plant Genetic Conservation Project under the royal initiation of Her Royal Highness Princess Maha Chakri Sirindhorn (RSPG) in 2010. The plant is rare, and its bioactive compounds and health-related information are unavailable. This is the first report on phenolic compositions, antioxidant activities, and in vitro health properties of this endangered species to provide information for the future development of functional food from *A. viridiflora* Craib. as well as establish a method for proper agricultural management. Once the bioactive compounds and health benefits of *A. viridiflora* Craib. are recognized, this endangered species will gain attention, leading to proper farming for commercial purposes. The long-term goal is to sustainably conserve the plant and appropriately utilize the potential benefits of *A. viridiflora* Craib. production. Results showed that (i) *A. viridiflora* Craib. is a rich source of naringenin, while other phenolics including apigenin, *p*-coumaric acid, sinapic acid, and caffeic acid were also detected; (ii) these phenolics led to high antioxidant activities detected in *A. viridiflora* Craib.; (iii) in vitro health properties targeting inhibition of the key enzymes controlling obesity (lipase), diabetes (α-glucosidase and dipeptidyl peptidase-IV), Alzheimer’s disease (cholinesterases and β-secretase), and hypertension (angiotensin-converting enzyme) might be the result of bioactivities of the phenolics detected in *A. viridiflora* Craib.

Bioactive compounds, including essential and non-essential compounds, are usually found in natural food such as vegetables, fruits, and whole grains [15,16]. These compounds can provide beneficial effects on health by behaving as antioxidants, inhibitors, or inducers of some diseases relevant to enzyme activity, inhibitors of receptor activities, and inhibitors or inducers of gene expression [17]. All of these biological functions can prevent many kinds of diseases, especially non-communicable diseases (NCDs). Nowadays, bioactive compounds are popularly used to maintain good health by focusing on healthy food. Copious research has investigated the bioactive compounds contained in many plants to determine their anti-inflammatory, anticancer, and antioxidant activities [18]. No details regarding the bioactive compounds of *A. viridiflora* Craib. are available, but other related species in the same genus were reported to contain some bioactive compounds. For example, leaves of *A. cissampeloides* contain tannins, saponins, phlobatannins, terpenoids, steroids, and alkaloids [19], while leaves of *A. lobata* contain catechic tannins, sterols/triterpenes, and alkaloids [20]. Other than leaves, roots of *A. hondala* contain *N*-acetyl galactosamine [21], while caudices of 10 Adenia species (*A. ellenbeckii*, *A. fruticosa*, *A. glauca*, *A. goetzii*, *A. keramanthus*, *A. lanceolata*, *A. racemosa*, *A. spinosa*, *A. stenodactyla*, and *A. venenata*) contain galactose-recognizing lectins [22]. Some of these lectins could strongly inhibit protein synthesis in cancer cells, leading to cell death and potential control of cancer [22]. Information on types and quantities of phenolic acids and flavonoids in Adenia spp. is not available, but it was previously suggested that *A. lobata* exhibited total phenolic contents (TPCs) of 3.60 mg gallic acid equivalent (GAE)/g dry weight (DW) [23], while *A. gummifera* exhibited 8.24 mg tannic acid equivalent (TAE)/g DW and 1.11 mg quercetin equivalent (QE)/g DW [24]. Our results showed TPCs ranging from 20.38 to 28.70 mg GAE/g DW for *A. viridiflora* Craib. that were 6–8 folds higher than *A. lobata*, while total flavonoid contents (TFCs) of *A. viridiflora* Craib. ranging from 5.96 to 14.08 mg QE/g DW were 5–13 fold higher than *A. gummifera*. This information suggested that species of plants greatly affect phenolic contents, while other external factors, i.e., extraction condition, growth location, and plant parts, also play significant roles in the amount of phenolics that can be extracted. Naringenin was the most abundant phenolic detected in *A. viridiflora* Craib., with contents insignificantly different among all plant areas, while TPCs and TFCs of all samples were similar. Since insignificantly different naringenin contents and low apigenin contents were detected in old leaves and young shoots, higher TFCs in old leaves most likely result from phenolic acid contents, with greater amounts detected in old leaves than young shoots. These results concurred with previous studies in *Aquilaria beccariana*, where older leaves were found to contain greater phenolic and flavonoid contents than younger leaves and shoots [25].

Phenolics are well-known as sources of antioxidants in plants. Many phenolics have been previously reported as strong antioxidants against free radicals and other reactive oxygen species (ROS) [26,27,28,29,30]. Naringenin, the most abundant phenolic detected in *A. viridiflora* Craib., was previously reported to possess high antioxidant activities with an oxygen radical antioxidant capacity (ORAC) value of 26,401.83 µmol Trolox equivalent (TE)/g, while high ORAC values of apigenin (29,623.84 µmol TE/g), sinapic acid (3363.15 µmol TE/g), and *p*-coumaric acid (24,382.80 µmol TE/g) were also reported [26,27,28,29,30]. No antioxidant activities have been reported in *A. viridiflora* Craib. but other related species in the same genus, including *A. lobata* recorded 2,2-diphenyl-1-picrylhydrazyl (DPPH) radical scavenging concentrations at 50% of 0.52 mg/mL [23], while *A. gummifera* had ferric reducing antioxidant power (FRAP) activities of 301.21 µmol Fe(II)/g, 2,2’-azino-bis(3-ethylbenzothiazoline-6-sulfonic acid (ABTS) activities of 94.2% inhibition at a concentration of 0.08 mg/mL, and DPPH free radical scavenging activity of 60% inhibition at a concentration of 0.1 mg/mL [24]. Corresponding to its high TPCs and TFCs, PN also exhibited higher antioxidant activities than plants from other areas. Old leaves exhibited high TPCs and TFCs, and possessed higher antioxidant activities than young shoots.

As well as being powerful antioxidants, phenolics are also effective inhibitors against key enzymes relevant to some NCDs, i.e., obesity, diabetes, Alzheimer’s disease (AD), and hypertension. Excessive energy intake, especially fat, is one of the main reasons for individuals having a high risk of obesity. Lipase is the key enzyme in fat digestion, and its inhibition can lead to low absorption of fatty acids into the body. It is possible that lipase inhibitory activities detected in *A. viridiflora* Craib. are the biological functions of phenolics that act as lipase inhibitors. The main phenolic in *A. viridiflora* Craib., naringenin, inhibited lipase with 10% inhibition at a concentration of 2200 µM, while other phenolics were reported to be even more effective inhibitors (caffeic acid with 35% inhibition at 10 µM, *p*-coumaric acid with 20% inhibition at 10 µM, sinapic acid with 10% inhibition at 10 µM, and apigenin with 50% inhibition at 800 µM) [31,32]. Even though a large amount of naringenin was present in *A. viridiflora* Craib., its low lipase inhibitory capacity resulted in low observed lipase inhibition.

To control diabetes, α-glucosidase is the key enzyme that converts polysaccharides into smaller subunits before absorption into the blood steam; thus, inhibition of α-glucosidase can decrease or delay a rise in blood glucose. Phenolics found in *A. viridiflora* Craib. were previously reported to possess α-glucosidase inhibitory activities. Naringenin is an effective α-glucosidase inhibitor with a half-maximal inhibitory concentration (IC_50_) of 0.075 mM, while apigenin, caffeic acid, sinapic acid, and *p*-coumaric acid are less effective, with higher IC_50_ values (0.2, 4.98, 6.1, and 30 mM, respectively) [33,34,35,36]. Targeting dipeptidyl peptidase-IV (DPP-IV) inhibition in diabetes leads to improvement of impaired insulin secretion, reduction of glucagon, and eventually lowering of bloodstream glucose. Interestingly, DPP-IV inhibitors in natural sources are mostly phenolics. Naringenin was found to be an effective inhibitor against DPP-IV with IC_50_ value of 0.24 µM, while apigenin and caffeic acid exhibited IC_50_ values of 0.14 and 3.37 µM, respectively [37,38]. Due to the effectiveness of phenolics against these enzymes, high inhibitory activities were observed in *A. viridiflora* Craib. extracts. Moreover, another related species in the same genus, *A. lobata*, was reported to lower blood glucose in streptozotocin-induced diabetic rats [23], suggesting the possibility that *Adenia* spp. could be used as a future antidiabetic agent.

The decrease in neurotransmitters by cholinesterases (ChEs) is one of the main hypotheses for AD pathology. Two ChEs, acetylcholinesterase (AChE) and butyrylcholinesterase (BChE), can break down neurotransmitters and acetylcholine (ACh), leading to a decline in brain function. Therefore, ChE inhibition could result in a slower rate of ACh degradation. Naringenin can act as an AChE inhibitor with an IC_50_ value of 143.6 µM, while apigenin (IC_50_ value of 81.5 µM), sinapic acid (75% inhibition at 5.2 mM), *p*-coumaric acid (50% inhibition at 5.2 mM), and caffeic acid (<50% inhibition at 5.2 mM) can also inhibit AChE with different degrees of inhibition [39,40,41]. These phenolics can also act as BChE inhibitors. Naringenin and apigenin were previously reported to exhibit IC_50_ values of 86.5 and 37.4 µM, respectively, against BChE, while lower inhibitory activities were detected in phenolic acids (caffeic acid with 20% inhibition, and sinapic acid and *p*-coumaric acid with <10% inhibition at the same concentration of 5.2 mM) [40,42]. Due to high IC_50_ values and low inhibition at high phenolic concentration, *A. viridiflora* Craib. extracts exhibited low AChE and BChE inhibitory activities. Another hypothesis of AD pathology is the induction of neuron damage by aggregated amyloid-beta (Aβ) peptide, produced from partial cleavage of the amyloid precursor protein (APP) by β-secretase or β-site amyloid precursor protein cleaving enzyme-1 (BACE-1). Therefore, inhibition of BACE-1 may lead to a delay in this process. Naringenin is able to inhibit BACE-1 with an IC_50_ value of 30.31 µM [43], while apigenin with IC_50_ value of 38.5 µM [44] and *p*-coumaric acid with IC_50_ value of 0.9 µM [45] are also strong BACE-1 inhibitors. No report of sinapic acid and caffeic acid on BACE-1 inhibitory activity is currently available; however, caffeic acid can improve spatial cognition and memory in vivo [46]. Due to the effectiveness of these phenolics, BACE-1 inhibitory activities were observed in *A. viridiflora* Craib. extracts were remarkably high.

Currently, angiotensin-converting enzyme (ACE) inhibitors are targeted as an alternative treatment of hypertension via the renin-angiotensin-aldosterone (RAAS) hormonal cascade. Naringenin with IC_50_ value of 0.196 mM is considered as a strong ACE inhibitor, while ACE inhibition was also observed with apigenin (IC_50_ value of 0.667 mM), caffeic acid (IC_50_ value of 2.1 mM), and *p*-coumaric acid (IC_50_ value of 2.8 mM) [47,48,49]. Other related species in the same genus as *A. viridiflora* Craib. have been found to have valuable properties, such as an aqueous extract of *A. cissampeloides*, which was found to reduce blood pressure, especially systolic blood pressure, in hypertensive subjects [50]. This information suggested that *A. viridiflora* Craib. showed potential as an antihypertensive agent.

In conclusion, *A. viridiflora* Craib. extracts were found to contain high phenolics, especially naringenin, antioxidant activities, and in vitro inhibitory activities of key enzymes that control the occurrence of obesity, diabetes, AD, and hypertension. Old leaves tended to exhibit higher phenolic and flavonoid contents, leading to greater antioxidant activities and in vitro enzyme inhibitory activities than young shoots. No clear trend that affected health-promoting bioactivities was observed in plants sourced from different areas (although PN and MN possessed higher inhibitory activities in most enzyme assays). However, these in vitro enzyme inhibitory activities were reported based on a single extract concentration, which was a limitation of our research—nevertheless, high inhibitory activities against α-glucosidase, DPP-IV, BACE-1, and ACE showed promising results. Therefore, further experiments to determine IC_50_ values of the plant extracts against these enzymes or in vivo experiments to confirm our preliminary results should be performed. Knowledge gained from this research can be used to promote agricultural information about *A. viridiflora* Craib. for the production and consumption as a plant with potential beneficial health properties against obesity, diabetes, AD, and hypertension.

## 4. Materials and Methods

### 4.1. Sample Collection, Preparation, and Extraction

The plants, *A. viridiflora* Craib., used in this study were collected from Kamphaeng Phet (KP), Muang Nakhon Ratchasima (MN), Pakchong Nakhon Ratchasima (PN), and Uthai Thani (UT), Thailand, and cultivated at the conservative plant areas for experimental botanical purposes at Khlong Phai, Sikhio district, Nakhon Ratchasima province, Thailand (14°86′18.7″ N and 101°56′83.3″ E). All plant samples were identified and authenticated by Renu Khumlert and Aschan Sukthumrong, Institute of Agricultural Technology, Suranaree University of Technology, Nakhon Ratchasima, Thailand. The voucher specimens, including BK No. 071408 (KP), BK No. 071410 (MN), BK No. 071411 (PN), and BK No. 071409 (UT), were deposited at the Bangkok Herbarium (BK), Bangkok, Thailand. The plant samples were harvested during March–April 2018. The parts of *A. viridiflora* Craib. were young shoots (length 30 cm from the top) and old leaves (length 30–50 cm from the top). The physical appearances and sizes of all samples were shown in Supplementary Table S1.

The samples were cleaned and freeze-dried using a freeze dryer (Heto PowerDry PL9000, Heto Lab Equipment, Allerod, Denmark) for 3 days. Dry samples were ground into fine powder using a grinder (Philips 600 W series, Philips Electronic Co., Ltd., Jakarta, Indonesia). The color of fresh and dried samples was examined using a ColorFlex EZ spectrophotometer (Hunter Associates Laboratory, Reston, VA, USA) and expressed as CIELAB units (L* representing dark (0) to white (100) colors, a* representing green (–) to red (+) colors, and b* representing blue (–) to yellow (+) colors, as shown in Supplementary Table S2). The moisture contents of fresh and dried samples were determined by a moisture analyzer (Halogen HE53 series, Mettler Toledo AG, Greifensee, Switzerland) and expressed as a percentage of moisture content, as shown in Supplementary Table S2. The powdery samples were kept at −20 °C until analysis.

The extraction of *A. viridiflora* Craib. was optimized as previously described [51]. Briefly, the powdery samples (0.5 g dry weight) were mixed with distilled water (10 mL) before incubating at 50 °C for 2 h using a temperature-controlled water bath shaker (a WNE45 series from Memmert GmBh, Eagle, WI, USA). The mixture was then centrifuged at 3800× *g* for 15 min using a refrigerated centrifuge (Hettich^®^ ROTINA 38R, Andreas Hettich GmbH, Tuttlingen, Germany). The supernatant was collected and filtered through a 0.45 µM polyethersulfone membrane syringe filter. The extracts were stored at −20 °C until analysis.

### 4.2. Determination of Phenolic Profiles

Phenolic profiles were analyzed using an Agilent 1100 HPLC system with a photodiode array detector and a 5 µm Zorbax Eclipse XDB-C_18_ column (150 × 4.6 mm) from Agilent Technologies (Santa Clara, CA, USA) as previously described [52]. Briefly, 0.5 g of dry sample was dissolved in 40 mL of 62.5% (*v*/*v*) aqueous methanol containing 0.5 g/L tBHQ and 10 mL of 6 N HCl. The mixture was incubated in a water bath shaker (WNE45 series, Memmert GmBh, Eagle, WI, USA) at 80 °C for 2 h before cooling in ice for 5 min. The mixture was then sonicated in an ultrasonic cleansing bath (Branson Ultrasonics™ M series, Branson Ultrasonics Corp., Danbury, CT, USA) for another 5 min and filtered through a polytetrafluoroethylene (0.22 µM) membrane syringe filter. The filtrate was injected into HPLC system with a gradient mobile phases consisting of Milli-Q water (18.2 MΩ.cm resistivity at 25 °C) containing 0.05% (*v*/*v*) TFA (solvent A), methanol containing 0.05% (*v*/*v*) TFA (solvent B), and acetonitrile containing 0.05% (*v*/*v*) TFA (solvent C) and a constant flow rate of 0.6 mL/min (Table 4). The existence of phenolic acids and flavonoids in the samples were analyzed by comparing retention time (*t_R_*) and spectral fingerprint with standards using the ChemStation software (Agilent Technologies, Santa Clara, CA, USA). Phenolic acid standards including 3,4-dihydroxybenzoic acid (≥97% T), chlorogenic acid (>98.0% HPLC, T), 4-hydroxybenzoic acid (>99.0% GC, T), caffeic acid (>98.0% HPLC, T), syringic acid (>97.0% T), *p*-coumaric acid (>98.0% GC, T), ferulic acid (>98.0% GC, T), and sinapic acid (>99.0% GC, T) were from Tokyo Chemical Industry Co. Ltd. (Tokyo, Japan), while gallic acid (97.5%–102.5% T), vanillic acid (≥97% HPLC), and rosmarinic acid (≥98% HPLC) were from Sigma-Aldrich (St. Louis, MO, USA). Flavonoid standards including hesperidin (>90.0% HPLC, T), myricetin (>97.0% HPLC), luteolin (>98.0% HPLC), quercetin (>98.0% HPLC, E), naringenin (>93.0% HPLC, T), kaempferol (>97.0% HPLC), apigenin (>98.0% HPLC), and genistein (>98.0% HPLC) were from Tokyo Chemical Industry Co. Ltd. (Tokyo, Japan), while isorhamnetin (≥99.0% HPLC) was from Extrasynthese (Genay, France) and rutin (≥94% HPLC) was from Sigma-Aldrich (St. Louis, MO, USA). The phenolic acids were visualized at 280 nm and 325 nm, while flavonoids were visualized at 338 nm and 368 nm. The HPLC chromatograms were shown in Supplementary Figures S1–S3.

Total phenolic contents (TPCs) were determined using Folin–Ciocalteu’s phenol reagent and a standard gallic acid (0–200 µg/mL) as previously described [53]. The presence of phenolics was detected on a Synergy™ HT 96-well UV-visible microplate reader (BioTek Instruments, Inc., Winooski, VT, USA) at 765 nm. The results were analyzed using a Gen 5 data analysis software (BioTek Instruments, Inc., Winooski, VT, USA) and expressed as mg gallic acid equivalent (GAE)/g dry weight (DW).

Total flavonoid contents (TFCs) were determined using an aluminum trichloride reagent and a standard quercetin (0–100 µg/mL), as previously described [54]. The presence of flavonoids was detected on the microplate reader at 510 nm. The results were analyzed using a Gen 5 data analysis software and expressed as mg quercetin equivalents (QE)/g DW.

### 4.3. Determination of Antioxidant Activities

Three methods for the determination of antioxidant activities were performed, including 2,2-diphenyl-1-picrylhydrazyl (DPPH) radical scavenging, ferric ion reducing antioxidant power (FRAP), and oxygen radical absorbance capacity (ORAC) assays as previously described [55]. The extract concentrations used in the antioxidant determination were chosen to be in the range of a standard curve.

In the DPPH radical scavenging assay, the extract (22 µL) was mixed with DPPH reagent (150 μM in 95% (*v*/*v*) aqueous ethanol, 200 µL) before incubating in dark at 25 °C for 30 min. The reaction was monitored at a wavelength of 520 nm using the microplate reader. Trolox (0.01–0.64 mM) was used as a standard.

The FRAP assay was determined using the extract (20 μL) and a prewarm (37 °C) FRAP reagent (10:1:1 ratio of 300 mM acetate buffer (pH 3.6), 10 mM 2,4,6-tri(2-pyridyl)-*S*-triazine in 40 mM HCl, and 20 mM FeCl_3_·6H_2_O solution, 150 μL). The mixture was incubated at 25 °C for 8 min. The reaction was monitored using the plate reader at a wavelength of 600 nm. Trolox (7.81–250.00 μM) was used as the standard.

For the ORAC assay, the extract (25 μL) was mixed with 80 nM sodium fluorescein (150 µL) before incubating at 37 °C for 30 min. To the reaction, 153 nM 2,2′-azobis(2-amidinopropane) dihydrochloride (25 µL) was added, and the fluorescence intensity was immediately monitored using an excitation wavelength of 485 nm and an emission wavelength of 528 nm. The results were calculated based on the differences in areas under the sodium fluorescein decay curve (AUC). The AUC was calculated as AUC = 0.5 + ƒ_1_/ƒ_0_ + ƒ_2_/ƒ_0_ + ƒ_3_/ƒ_0_ +…+ (0.5) ƒ_90_/ƒ_0_, where ƒ_0_ is the initiation fluorescence reading at 0 min, and ƒ_i_ is the fluorescence reading at i min. Trolox (3.12–100.00 μM) was used as the standard.

### 4.4. Enzyme Inhibitory Activities

The lipase inhibitory activity was determined using *Candida rugosa* lipase (type VII, ≥700 unit/mg, 100 µL of 0.01 mg/mL) as an enzyme, 5-5’-dithiobis(2-nitrobenzoic-*N*-phenacyl-4,5-dimethyyhiazolium bromide) (50 µL of 0.2 mM) as a substrate, 5,5′-dithiobis(2-nitrobenzoic acid) (DTNB, 10 µL of 16 mM) as an indicator, and a plant extract (40 µL) as an inhibitor as previously described [56]. The inhibitory activity was monitored at 412 nm using the microplate reader, and the results were expressed as the inhibition percentage using the following equation

$$\%\;\mathrm{inhibition}\;=\;\left(1\;-\;\frac{B-b}{A-a}\right)\;\times \;100,$$

where *A* is the initial velocity of the control reaction with an enzyme (control), *a* is the initial velocity of the control reaction without enzyme (control blank), *B* is the initial velocity of the enzyme reaction with extract (sample), and *b* is the initial velocity of the reaction with extract but without enzyme (sample blank).

The α-glucosidase inhibitory activity was determined using *Saccharomyces cerevisiae* α-glucosidase (type 1, ≥10 U/mg protein, 100 µL of 0.1 U/mL) as an enzyme, *p*-nitrophenyl-α-D-glucopyranoside (50 µL of 2 mM) as a substrate and an indicator, and a plant extract (50 µL) as an inhibitor as previously described [56]. The inhibitory activity was monitored at 405 nm using the microplate reader, and the results were expressed as the inhibition percentage as above.

The dipeptidyl peptidase-IV (DPP-IV) inhibitory activity was determined as previously described [57] with some modification as follows. Briefly, the assay consisted of human dipeptidyl peptidase-IV (recombinant, expressed in baculovirus infected *Sf*9 cells, ≥10 units/mg, 50 µL of 0.02 U/mL), Gly-Pro-*p*-nitroanilide hydrochloride (H-Gly-Pro-pNA·HCl, 25 µL of 12 mM) in 100 mM Tris-HCl (pH 8.0), Tris-HCl (pH 8.0, 100 µL of 100 mM), and sample extract (25 µL). The DPP-IV inhibitory activity was monitored at 405 nm using the microplate reader, and the results were expressed as the inhibition percentage as above.

The acetylcholinesterases (AChE) inhibitory activity was determined using *Electrophorus electricus* AChE (1000 units/mg, 100 μL of 20 ng) as an enzyme, acetylthiocholine (40 μL of 0.8 mM) as a substrate, DTNB (10 μL of 16 mM) as an indicator, and a plant extract (40 µL) as an inhibitor as previously described [56]. The inhibitory activity was monitored at 412 nm using the microplate reader, and the results were expressed as the inhibition percentage as above.

The butyrylcholinesterases (BChE) inhibitory activity was determined using equine serum BChE (≥10 units/mg protein, 100 µL of 0.5 µg/mL) as an enzyme, butyrylthiocholine (50 µL of 0.4 mM) as a substrate, DTNB (10 μL of 16 mM) as an indicator, and a plant extract (40 µL) as an inhibitor as previously described [56]. The inhibitory activity was monitored at 412 nm using the microplate reader, and the results were expressed as the inhibition percentage as above.

The β-secretase (BACE-1) inhibitory activity was determined using a BACE-1 FRET assay kit (Sigma-Aldrich, St. Louis, MO, USA). The inhibitory activity was monitored with an excitation wavelength of 320 nm and an emission wavelength of 405 nm using the microplate reader, and the results were expressed as the inhibition percentage using the following equation

$$\%\;\mathrm{inhibition}\;=\;\left(1\;-\;\frac{B-b}{A-a}\right)\;\times \;100,$$

where *A* is the absorbance of the control reaction with an enzyme (control), *a* is the absorbance of the control reaction without enzyme (control blank), *B* is the absorbance of the enzyme reaction with extract (sample), and *b* is the absorbance of the reaction with extract but without enzyme (sample blank).

The angiotensin-converting enzyme (ACE) inhibitory activity was determined using rabbit lung angiotensin-converting enzyme (≥2 unit/mg, 3 µL of 0.5 U/mL) as an enzyme, hippuryl-histidyl-leucine (HHL, 30 µL of 3 mM) as a substrate, *o*-phthaldialdehyde (15 µL of 20 mg/mL) as an indicator, and a plant extract (50 µL) as an inhibitor, as previously described [56]. The inhibitory activity was monitored with an excitation wavelength of 360 nm and an emission wavelength of 485 nm using the microplate reader, and the results were expressed as the inhibition percentage, similar to the BACE-1 assay.

All the enzymes, chemicals, and reagents in the enzyme inhibitory assays were purchased from Sigma-Aldrich (St. Louis, MO, USA).

### 4.5. Statistical Analysis

All experiments were performed in triplicate (*n* = 3) and present as mean ± standard deviation (SD). The significant differences between values with *p* < 0.05 were determined using one-way Analysis of Variance (ANOVA) followed by a Duncan’s multiple comparison test and unpaired *t*-test.

## Figures and Tables

**Table 1:** Phenolic profiles, total phenolic contents (TPCs), and total flavonoid contents (TFCs) in old leaves and young shoots of the four *Adenia viridiflora* Craib. plant sources.

*A. viridiflora* Craib. Sources	Phenolics (mg/100 g DW)					TPCs (mg GAE/g DW)	TFCs (mg QE/g DW)
	Phenolic Acids			Flavonoids			
	Caffeic Acid	*p*-Coumaric Acid	Sinapic Acid	Naringenin	Apigenin		
**Old leaves**							
KP	7.78 ± 0.16 ^b^	78.44 ± 4.32 ^b^*	8.66 ± 0.44 ^c^*	2055.79 ± 50.36 ^a^	2.89 ± 0.52 ^b^*	28.70 ± 1.79 ^a^*	5.96 ± 0.35 ^c^*
MN	ND	97.09 ± 2.88 ^a^*	10.31 ± 0.18 ^b^*	1982.69 ± 102.84 ^a^	10.60 ± 3.66 ^a^*	25.37 ± 1.16 ^b^	8.35 ± 0.61 ^b^*
PN	ND	55.86 ± 3.61 ^c^*	14.00 ± 1.28 ^a^*	1957.50 ± 122.01 ^a^	6.09 ± 0.28 ^ab^*	27.89 ± 1.61 ^a^*	14.08 ± 1.41 ^a^*
UT	11.79 ± 0.92 ^a^*	79.20 ± 4.31 ^b^*	8.60 ± 0.61 ^c^*	1968.48 ± 81.76 ^a^	2.03 ± 2.22 ^b^	23.56 ± 1.34 ^c^*	8.38 ± 0.33 ^b^*
**Young shoots**							
KP	ND	25.22 ± 2.56 ^b^	4.79 ± 0.01 ^b^	2164.95 ± 108.27 ^a^	11.82 ± 2.11 ^a^	20.57 ± 0.72 ^c^	7.35 ± 0.42 ^b^
MN	ND	38.98 ± 0.37 ^a^	7.19 ± 0.49 ^a^	2127.74 ± 66.19 ^a^	2.89 ± 1.77 ^b^	26.35 ± 1.84 ^a^	7.15 ± 0.50 ^b^
PN	ND	22.60 ± 0.93 ^b^	6.52 ± 0.02 ^a^	2100.10 ± 36.02 ^a^	1.22 ± 0.98 ^b^	23.57 ± 0.92 ^b^	9.65 ± 0.72 ^a^
UT	2.21 ± 0.80	23.43 ± 1.42 ^b^	4.49 ± 0.26 ^b^	2054.07 ± 38.36 ^a^	5.22 ± 0.25 ^b^	20.38 ± 0.79 ^c^	7.50 ± 0.69 ^b^

Values are expressed as mean ± standard deviation (SD) of triplicate experiments (*n* = 3). DW: dry weight; GAE: gallic acid equivalent; QE: quercetin equivalent; ND: not detected; KP: Kamphaeng Phet origin; MN: Muang Nakhon Ratchasima origin; PN: Pakchong Nakhon Ratchasima origin; UT: Uthai Thani origin; different lower case letters indicate significant differences at *p* < 0.05 of the same phenolics in the same plant part of plants from different origins using one-way analysis of variance (ANOVA) and Duncan’s multiple comparison test; * indicates significant difference (*p* < 0.05) of the same phenolic in old leaves and young shoots of plants originating from the same area using the unpaired *t*-test.

**Table 2:** Antioxidant activities in old leaves and young shoots of the four *A. viridiflora* Craib. plant sources.

*A. viridiflora* Craib. Sources	Antioxidant Activities		
	DPPH Radical Scavenging Assay (µmol TE/100 g DW)	FRAP Assay (µmol TE/g DW)	ORAC Assay (µmol TE/g DW)
**Old leaves**			
KP	0.96 ± 0.07 ^b^*	34.08 ± 3.12 ^b^*	753.77 ± 74.81 ^c^*
MN	0.99 ± 0.06 ^b^*	35.74 ± 3.27 ^ab^*	1094.66 ± 119.33 ^b^*
PN	1.44 ± 0.10 ^a^*	37.00 ± 1.60 ^a^*	1403.53 ± 122.44 ^a^
UT	0.97 ± 0.05 ^b^*	35.17 ± 2.36 ^ab^*	1106.77 ± 102.36 ^b^
**Young shoots**			
KP	1.25 ± 0.06 ^b^	16.52 ± 0.97 ^b^	1148.47 ± 74.70 ^b^
MN	1.37 ± 0.04 ^a^	29.24 ± 2.88 ^a^	842.36 ± 69.59 ^c^
PN	1.28 ± 0.02 ^b^	28.03 ± 0.98 ^a^	1438.85 ± 145.27 ^a^
UT	1.23 ± 0.10 ^b^	14.36 ± 0.85 ^c^	1066.29 ± 100.98 ^b^

Values are expressed as mean ± standard deviation (SD) of triplicate experiments (*n* = 3). TE: Trolox equivalent; DW: dry weight; KP: Kamphaeng Phet origin; MN: Muang Nakhon Ratchasima origin; PN: Pakchong Nakhon Ratchasima origin; UT: Uthai Thani origin; different lower case letters indicate significant differences at *p* < 0.05 of the same antioxidant measurements in the same plant part of plants from different areas of origin using one-way analysis of variance (ANOVA) and Duncan’s multiple comparison test; * indicates significant difference (*p* < 0.05) of the same antioxidant measurement in old leaves and young shoots of plants originating from the same areas using the unpaired *t*-test. DPPH: 2,2-diphenyl-1-picrylhydrazyl; FRAP: ferric reducing antioxidant power; ORAC: oxygen radical antioxidant capacity.

**Table 3:** Inhibitory activities in old leaves and young shoots of the four *A. viridiflora* Craib. plant Scheme 1. and hypertension (angiotensin-converting enzyme (ACE)).

*A. viridiflora* Craib. Sources	Inhibitory Activity (%)						
	Obesity	Diabetes		Alzheimer’s Disease			Hypertension
	^1^ Lipase	^2^ α-Glucosidase	^3^ DPP-IV	^1^ AChE	^1^ BChE	^4^ BACE-1	^5^ ACE
**Old leaves**							
KP	14.81 ± 1.30 ^c^*	58.08 ± 5.26 ^c^*	46.07 ± 4.55 ^c^*	27.70 ± 2.12 ^a^	8.91 ± 0.65 ^d^*	78.57 ± 1.49 ^bc^	48.58 ± 3.97 ^b^
MN	23.54 ± 1.32 ^a^*	66.48 ± 5.43 ^b^	61.96 ± 3.36 ^b^*	20.80 ± 1.43 ^c^	18.70 ± 1.87 ^a^*	85.01 ± 6.82 ^ab^	60.33 ± 5.84 ^a^*
PN	22.30 ± 1.65 ^a^*	75.09 ± 4.84 ^a^*	69.89 ± 4.47 ^a^*	23.91 ± 1.40 ^b^*	13.03 ± 1.01 ^c^*	90.63 ± 3.17 ^a^*	61.71 ± 6.03 ^a^
UT	20.53 ± 1.75 ^b^*	62.39 ± 3.57 ^bc^*	62.35 ± 6.27 ^b^*	22.59 ± 1.59 ^b^	14.65 ± 1.23 ^b^*	72.60 ± 0.70 ^c^	52.73 ± 4.92 ^b^
**Young shoots**							
KP	10.16 ± 0.98 ^c^	79.87 ± 6.14 ^a^	30.19 ± 3.07 ^d^	28.68 ± 2.52 ^a^	7.18 ± 0.85 ^c^	81.97 ± 4.52 ^a^	47.84 ± 4.74 ^d^
MN	17.24 ± 1.51 ^a^	66.15 ± 3.94 ^b^	74.14 ± 2.88 ^a^	22.13 ± 2.20 ^b^	15.02 ± 1.06 ^a^	72.13 ± 4.68 ^b^	65.53 ± 1.39 ^a^
PN	12.41 ± 1.11 ^b^	82.82 ± 5.98 ^a^	54.01 ± 4.89 ^b^	16.29 ± 1.51 ^c^	5.18 ± 0.74 ^d^	78.57 ± 4.47 ^ab^	59.65 ± 1.31 ^b^
UT	8.41 ± 0.79 ^d^	68.69 ± 5.38 ^b^	44.74 ± 4.05 ^c^	21.94 ± 1.76 ^b^	9.69 ± 0.81 ^b^	70.49 ± 0.99 ^b^	54.36 ± 3.31 ^c^

Values are expressed as mean ± standard deviation (SD) of triplicate experiments (*n* = 3). KP: Kamphaeng Phet origin; MN: Muang Nakhon Ratchasima origin; PN: Pakchong Nakhon Ratchasima origin; UT: Uthai Thani origin; different lower case letters indicate significant differences at *p* < 0.05 of the same enzyme inhibitory activities in the same plant part of plants originating from different areas using one-way analysis of variance (ANOVA) and Duncan’s multiple comparison test; * indicates significant difference (*p* < 0.05) between old leaves and young shoots of plants originating from the same area using the unpaired *t*-test. ^1^ Final concentration = 10.00 mg/mL; ^2^ final concentration = 12.50 mg/mL; ^3^ final concentration = 1.25 mg/mL; ^4^ final concentration = 1.50 mg/mL; ^5^ final concentration = 0.08 mg/mL.

**Table 4:** Solvent system for high performance liquid chromatography (HPLC) analysis to identify phenolics.

Time (min)	Flow Rate (mL/min)	Solvent A (%)	Solvent B (%)	Solvent C (%)
0	0.6	90	6	4
5	0.6	85	9	6
30	0.6	71	17.4	11.6
60	0.6	0	85	15
61	0.6	90	6	4
66	0.6	90	6	4

Solvent A = Milli-Q water containing 0.05% (*v*/*v*) TFA; solvent B = methanol containing 0.05% (*v*/*v*) TFA; solvent C = acetonitrile containing 0.05% (*v*/*v*) TFA.

## Data Availability

Data is contained within this article and Supplementary Materials.

## Supplementary Materials

The following are available online, Table S1: Images of old leaves and young shoots of Kamphaeng Phet (KP), Muang Nakhon Ratchasima (MN), Pakchong Nakhon Ratchasima (PN), and Uthai Thani (UT) originated *Adenia viridiflora* Craib., Table S2: Color (where L* describes darkness (−) to lightness (+), a* describes green (−) to red (+) colors, and b* describes indigo (−) to yellow (+)) and the percentage (%) of moisture content of fresh and dried old leaves and young shoots of Kamphaeng Phet (KP), Muang Nakhon Ratchasima (MN), Pakchong Nakhon Ratchasima (PN), and Uthai Thani (UT) originated *Adenia viridiflora* Craib., Figure S1: High performance liquid chromatograms of standards including (A.) caffeic acid, (B.) *p*-coumaric acid, and (C.) sinapic acid, and samples including old leaves of (D.) Kamphaeng Phet (KP), (E.) Muang Nakhon Ratchasima (MN), (F.) Pakchong Nakhon Ratchasima (PN), and (G.) Uthai Thani (UT) and young shoots of (H.) KP, (I.) MN, (J.) PN, and (K.) UT originated *Adenia viridiflora* Craib. Retention times (*R_t_*) of phenolics are indicated at a wavelength of 325 nm., Figure S2: High performance liquid chromatograms of standards including (A.) naringenin and samples including old leaves of (B.) Kamphaeng Phet (KP), (C.) Muang Nakhon Ratchasima (MN), (D.) Pakchong Nakhon Ratchasima (PN), and (E.) Uthai Thani (UT) and young shoots of (F.) KP, (G.) MN, (H.) PN, and (I.) UT originated *Adenia viridiflora* Craib. Retention times (*R_t_*) of phenolics are indicated at a wavelength of 280 nm., Figure S3: High performance liquid chromatograms of standards including (A.) apigenin and samples including old leaves of (B.) Kamphaeng Phet (KP), (C.) Muang Nakhon Ratchasima (MN), (D.) Pakchong Nakhon Ratchasima (PN), and (E.) Uthai Thani (UT) and young shoots of (F.) KP, (G.) MN, (H.) PN, and (I.) UT originated *Adenia viridiflora* Craib. Retention times (*R_t_*) of phenolics are indicated at a wavelength of 338 nm.

## Sample Availability

Samples of the compounds are not available from the authors.
