# Supplementary material for: In Vitro Phytotherapeutic Properties of Aqueous Extracted Adenia viridiflora Craib. towards Civilization Diseases
Source: Molecules. 2021 Feb 18;26(4):1082. doi: 10.3390/molecules26041082 (PMC7922288; doi:10.3390/molecules26041082)

molecules

MDPI

Supplementary materials

In Vitro Phytotherapeutic Properties of Aqueous Extracted *Adenia viridiflora* Craib. towards Civilization Diseases

Werawat Wannasaksri [1], Nattira On–Nom [1,2], Chaowanee Chupeerach [1,2], Piya Temviriyanukul [1,2], Somsri Charoenkiatkul [1] and Uthaiwan Suttisansanee [1,2,*]

1. Institute of Nutrition, Mahidol University, Salaya, Phuttamonthon, Nakhon Pathom 73170, Thailand; nit.frank@gmail.com (W.W.); nattira.onn@mahidol.ac.th (N.O.); chaowanee.chu@mahidol.ac.th (C.C.); piya.tem@mahidol.ac.th (P.T.); somsri.chr@mahidol.ac.th (S.C.); uthaiwan.sut@mahidol.ac.th (U.S.)
2. Food and Nutrition Academic and Research Cluster, Institute of Nutrition, Mahidol University, Salaya, Phuttamonthon, Nakhon Pathom 73170, Thailand

* Correspondence: uthaiwan.sut@mahidol.ac.th; Tel.: +662-800-2380 ext. 422

Supplementary Table S1:

Images of old leaves and young shoots of Kamphaeng Phet (KP), Muang Nakhon Ratchasima (MN), Pakchong Nakhon Ratchasima (PN), and Uthai Thani (UT) originated *Adenia viridiflora* Craib.

Origins	Plant parts	
	Old leaves	Young shoots
Kamphaeng Phet (KP)	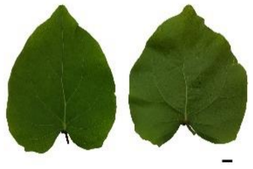	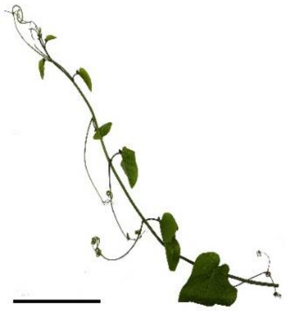
Muang Nakhon Ratchasima (MN)	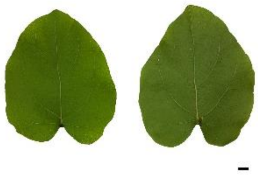	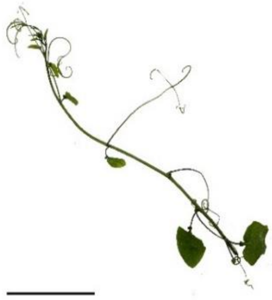
Pakchong Nakhon Ratchasima (PN)	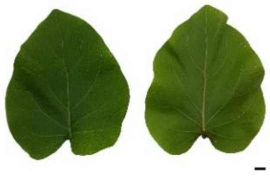	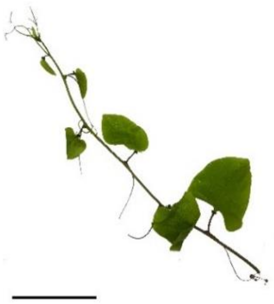
Uthai Thani (UT)	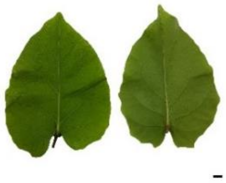	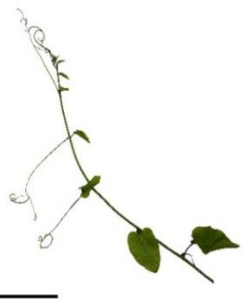

Note: − scale of 1 cm for old leaves and of 10 cm for young shoots.

Supplementary Table S2:

Color (where L* describes darkness (−) to lightness (+), a* describes green (−) to red (+) colors, and b* describes indigo (−) to yellow (+)) and the percentage (%) of moisture content of fresh and dried old leaves and young shoots of Kamphaeng Phet (KP), Muang Nakhon Ratchasima (MN), Pakchong Nakhon Ratchasima (PN), and Uthai Thani (UT) originated *Adenia viridiflora* Craib.

Varieties	Color value			Moisture content (%)
	L*	a*	b*	
**Fresh old leaves**				
– Kamphaeng Phet	42.92 ± 1.53	–9.66 ± 0.73	25.38 ± 1.11	80.17 ± 0.17
– Muang, Nakhon Ratchasima	47.00 ± 2.39	–9.47 ± 0.02	26.94 ± 2.05	80.43 ± 0.67
– Pakchong, Nakhon Ratchasima	46.12 ± 0.55	–10.02 ± 0.20	28.12 ± 1.47	82.11 ± 0.51
– Uthai Thani	46.80 ± 3.80	–9.29 ± 0.51	29.65 ± 1.42	80.91 ± 0.57
**Dried old leaves**				
– Kamphaeng Phet	35.37 ± 0.52	–5.14 ± 0.08	14.61 ± 0.41	6.19 ± 0.54
– Muang, Nakhon Ratchasima	35.17 ± 0.98	–5.19 ± 0.21	14.85 ± 0.80	6.77 ± 0.23
– Pakchong, Nakhon Ratchasima	35.48 ± 1.19	–4.81 ± 1.07	14.36 ± 1.75	6.71 ± 0.26
– Uthai Thani	32.34 ± 0.11	–3.87 ± 0.06	11.95 ± 0.04	7.81 ± 1.40
**Fresh young shoots**				
– Kamphaeng Phet	34.50 ± 1.70	–6.49 ± 0.89	22.52 ± 1.07	85.78 ± 0.40
– Muang, Nakhon Ratchasima	32.27 ± 4.61	–5.92 ± 0.89	21.51 ± 1.50	83.99 ± 0.37
– Pakchong, Nakhon Ratchasima	41.89 ± 1.43	–8.87 ± 0.57	26.71 ± 1.91	85.47 ± 0.30
– Uthai Thani	35.96 ± 4.69	–7.19 ± 2.95	24.60 ± 4.40	86.87 ± 0.77
**Dried young shoots**				
– Kamphaeng Phet	36.86 ± 0.63	–4.97 ± 0.27	14.07 ± 0.84	6.79 ± 0.83
– Muang, Nakhon Ratchasima	37.48 ± 0.26	–5.02 ± 0.02	14.79 ± 0.35	5.39 ± 0.95
– Pakchong, Nakhon Ratchasima	39.05 ± 0.43	–5.52 ± 0.10	16.82 ± 0.23	7.79 ± 0.31
– Uthai Thani	38.90 ± 0.17	–4.90 ± 0.13	15.07 ± 0.36	6.73 ± 2.30

All data were expressed as mean ± standard deviation (SD) of triplicate experiments (n = 3).

Supplementary Figure 1:

High performance liquid chromatograms of standards including (A.) naringenin and samples including old leaves of (B.) Kamphaeng Phet (KP), (C.) Muang Nakhon Ratchasima (MN), (D.) Pakchong Nakhon Ratchasima (PN), and (E.) Uthai Thani (UT) and young shoots of (F.) KP, (G.) MN, (H.) PN, and (I.) UT originated *Adenia viridiflora* Craib. Retention times (R_t) of phenolics are indicated at a wavelength of 280 nm.

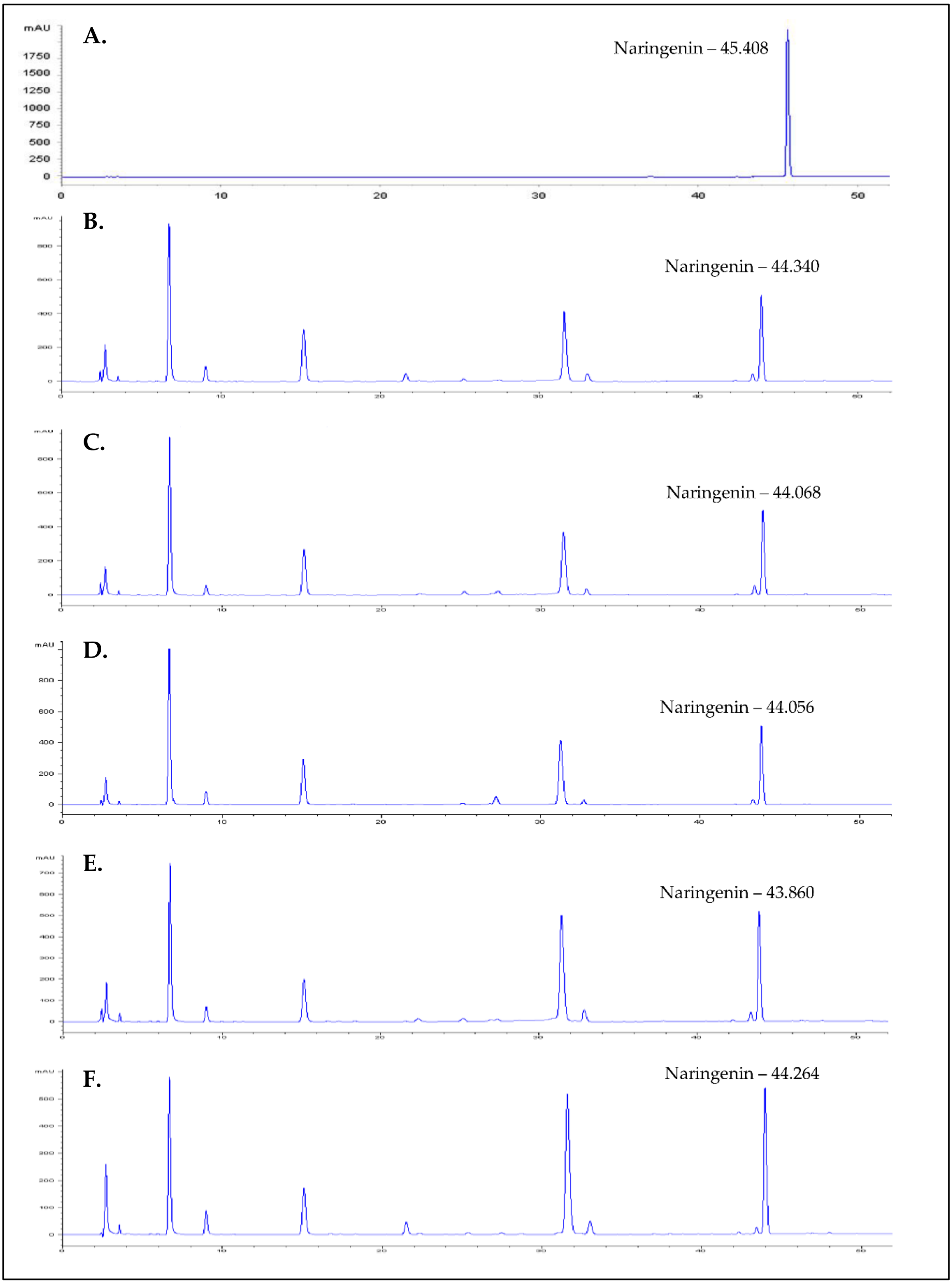

Supplementary Figure 1 (Cont.):

High performance liquid chromatograms of standards including (A.) naringenin and samples including old leaves of (B.) Kamphaeng Phet (KP), (C.) Muang Nakhon Ratchasima (MN), (D.) Pakchong Nakhon Ratchasima (PN), and (E.) Uthai Thani (UT) and young shoots of (F.) KP, (G.) MN, (H.) PN, and (I.) UT originated *Adenia viridiflora* Craib. Retention times (R_t) of phenolics are indicated at a wavelength of 280 nm.

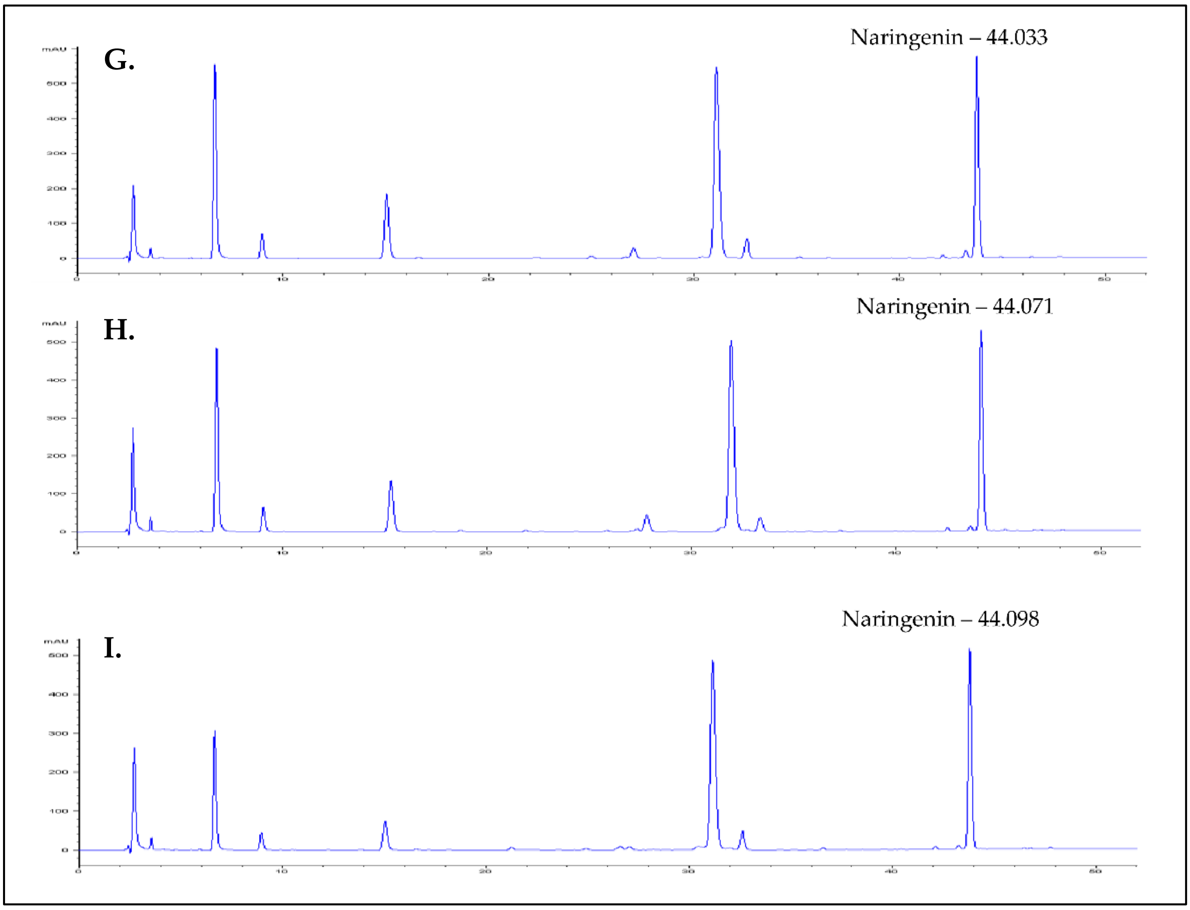

Supplementary Figure S2:

High performance liquid chromatograms of standards including (A.) caffeic acid, (B.) *p*-coumaric acid, and (C.) sinapic acid, and samples including old leaves of (D.) Kamphaeng Phet (KP), (E.) Muang Nakhon Ratchasima (MN), (F.) Pakchong Nakhon Ratchasima (PN), and (G.) Uthai Thani (UT) and young shoots of (H.) KP, (I.) MN, (J.) PN, and (K.) UT originated *Adenia viridiflora* Craib. Retention times (R_t) of phenolics are indicated at a wavelength of 325 nm.

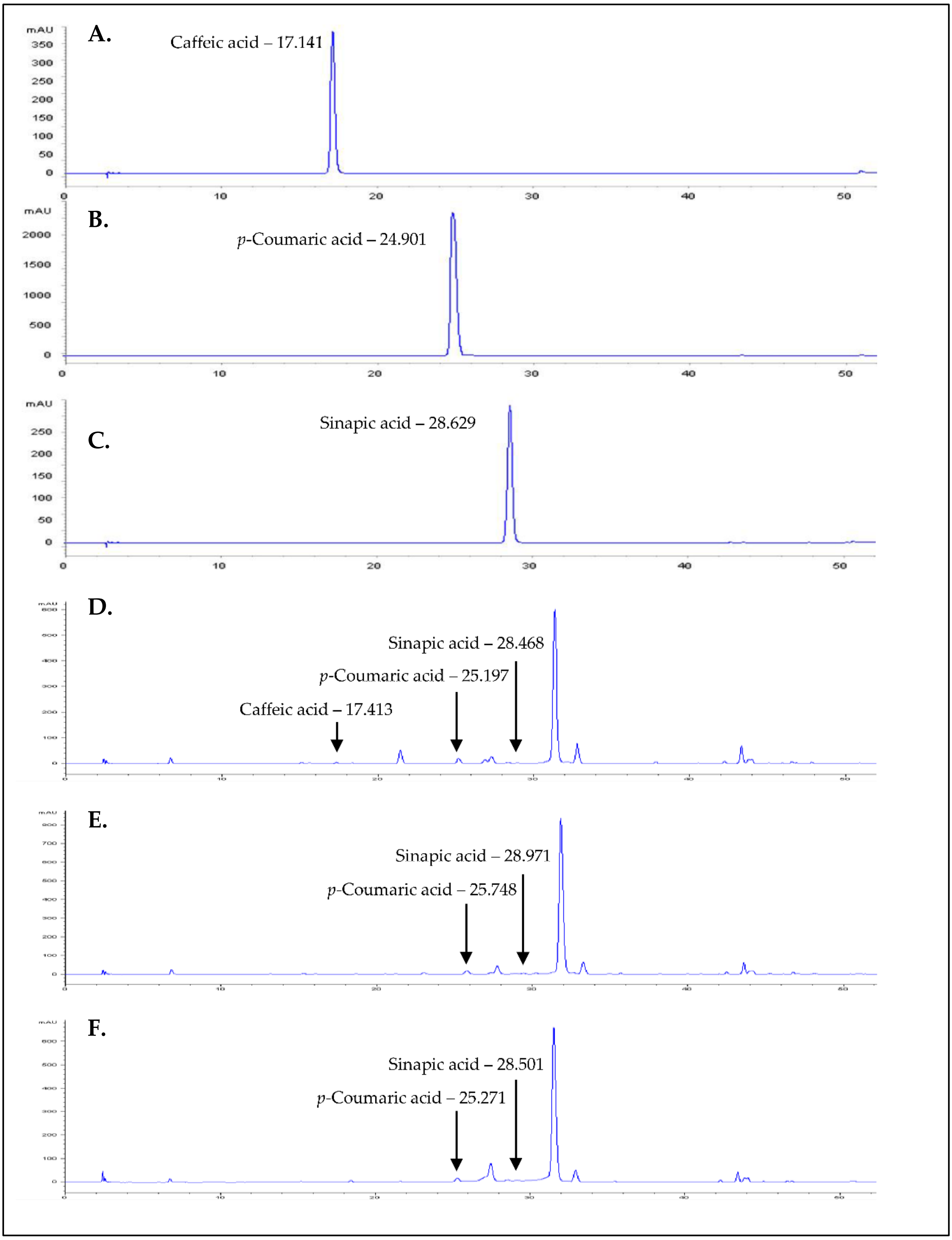

Supplementary Figure S2 (Cont.):

High performance liquid chromatograms of standards including (A.) caffeic acid, (B.) *p*-coumaric acid, and (C.) sinapic acid, and samples including old leaves of (D.) Kamphaeng Phet (KP), (E.) Muang Nakhon Ratchasima (MN), (F.) Pakchong Nakhon Ratchasima (PN), and (G.) Uthai Thani (UT) and young shoots of (H.) KP, (I.) MN, (J.) PN, and (K.) UT originated *Adenia viridiflora* Craib. Retention times (R_t) of phenolics are indicated at a wavelength of 325 nm.

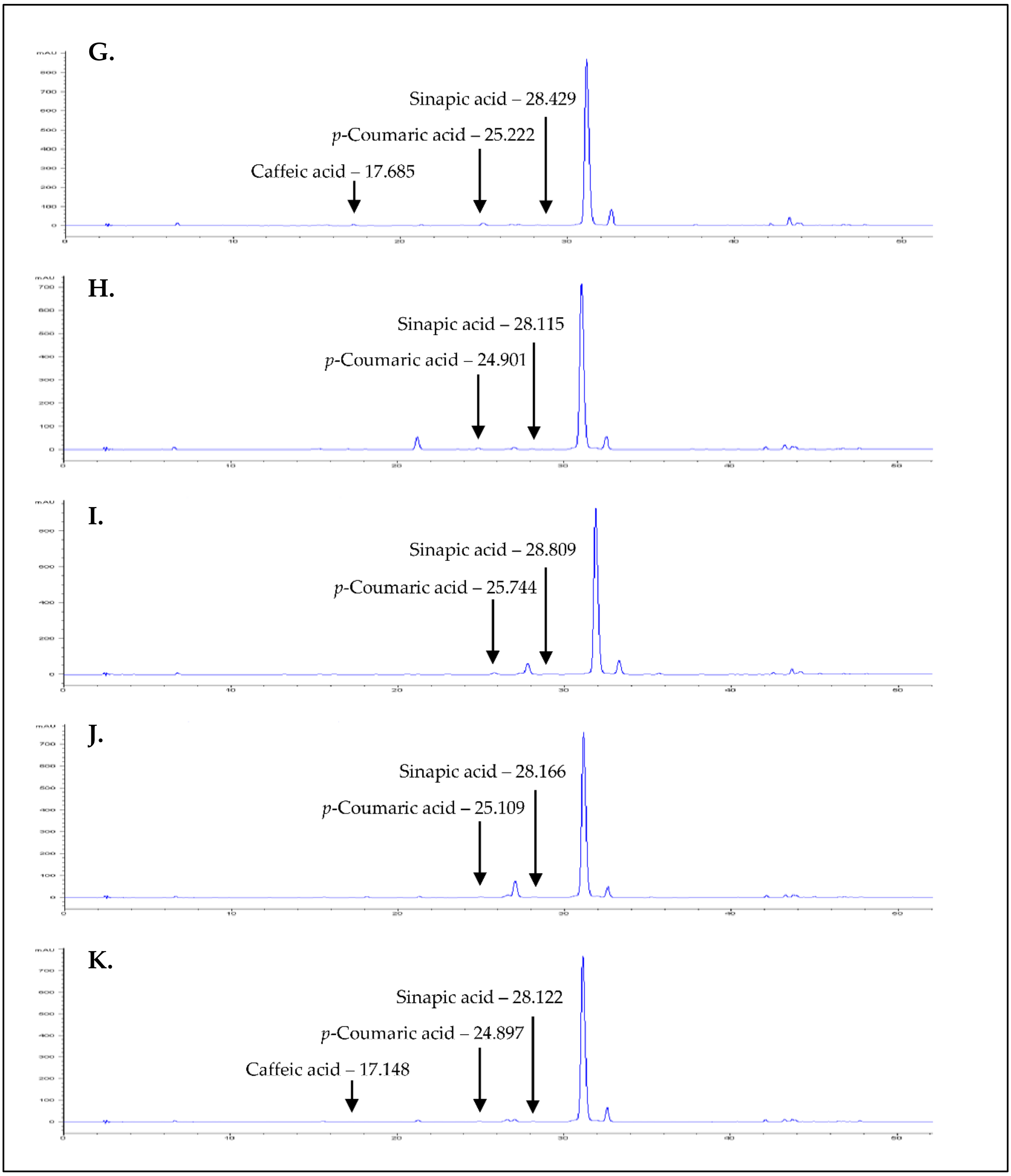

Supplementary Figure S3:

High performance liquid chromatograms of standards including (A.) apigenin and samples including old leaves of (B.) Kamphaeng Phet (KP), (C.) Muang Nakhon Ratchasima (MN), (D.) Pakchong Nakhon Ratchasima (PN), and (E.) Uthai Thani (UT) and young shoots of (F.) KP, (G.) MN, (H.) PN, and (I.) UT originated *Adenia viridiflora* Craib. Retention times (R_t) of phenolics are indicated at a wavelength of 338 nm.

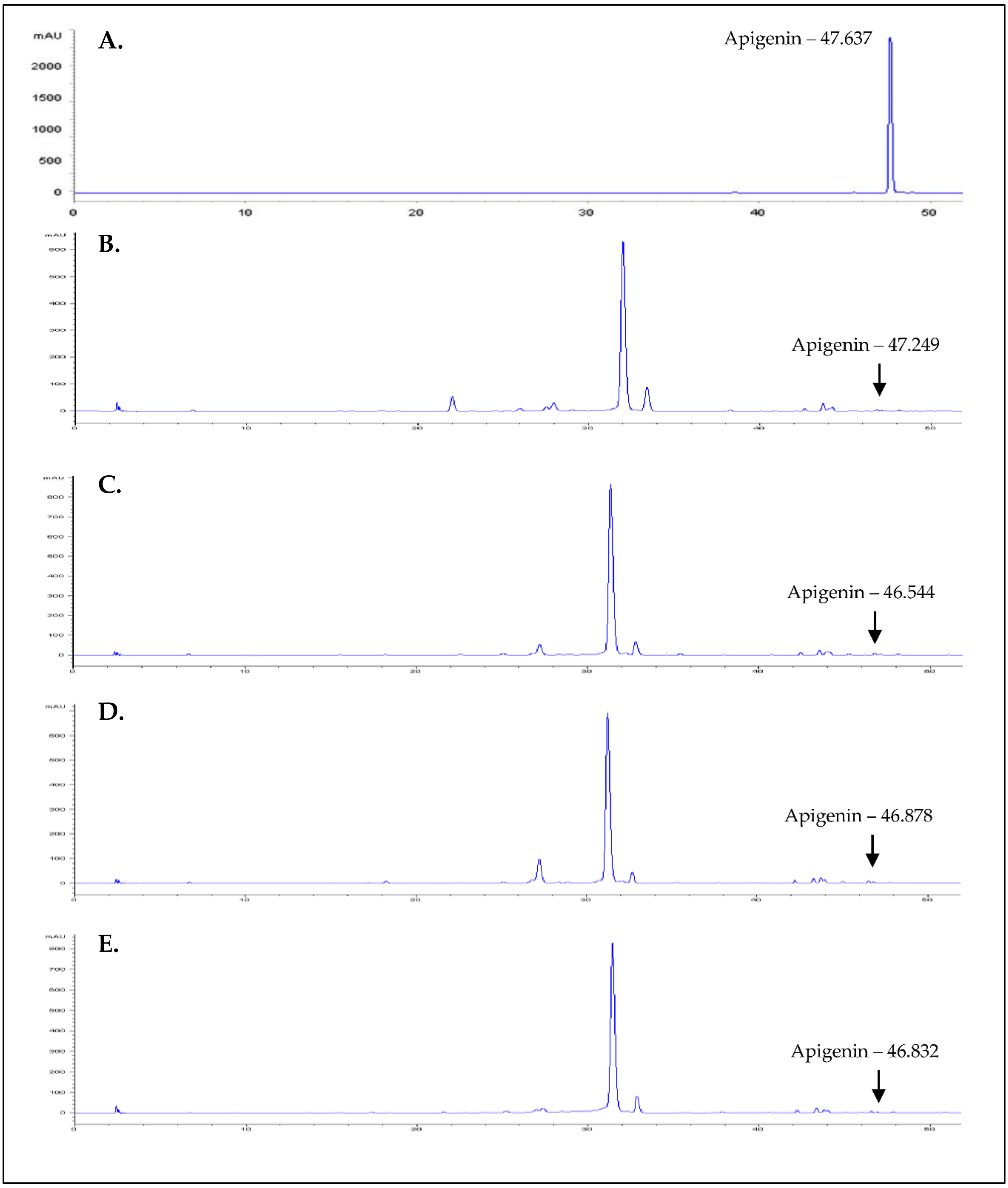

Supplementary Figure S3 (Cont.):

High performance liquid chromatograms of standards including (A.) apigenin and samples including old leaves of (B.) Kamphaeng Phet (KP), (C.) Muang Nakhon Ratchasima (MN), (D.) Pakchong Nakhon Ratchasima (PN), and (E.) Uthai Thani (UT) and young shoots of (F.) KP, (G.) MN, (H.) PN, and (I.) UT originated *Adenia viridiflora* Craib. Retention times (R_t) of phenolics are indicated at a wavelength of 338 nm.

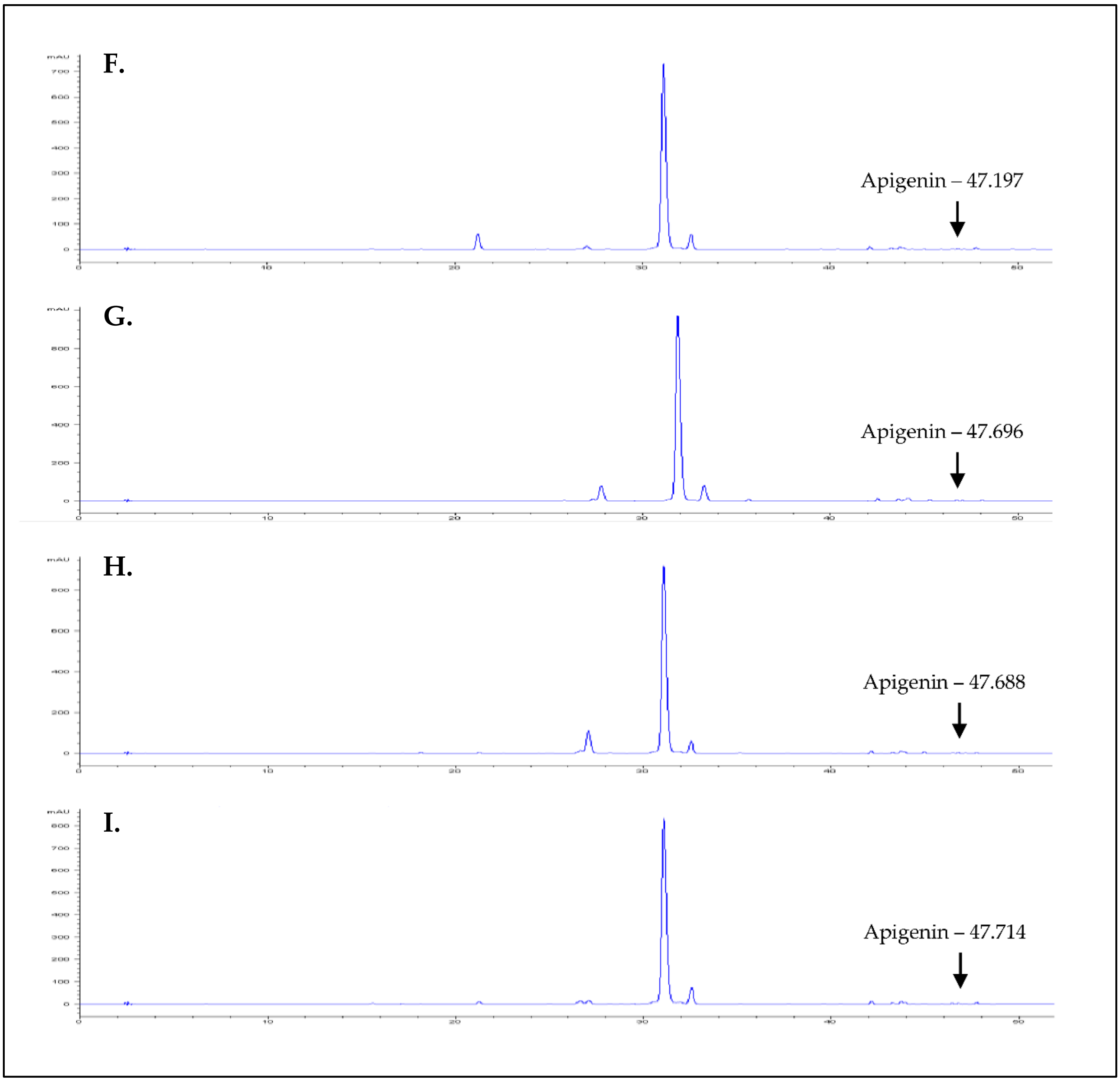

Supplement: Supplementary file 1 [file molecules-26-01082-s001.pdf]
